# Below Par

**Published:** 1889-10-05

**Authors:** 


### The Doctor's Pulpit.

### "BELOW PAR."

The "uncomfortableness" of the condition which is commonly described by the words "below par," is what most strikes the man who is the victim of it. If it be his clothes that are below par he is painfully conscious of the "seediness" and "shabbiness" of his *tout ensemble*. The most industrious brushing will not make an old hat new, though a warmed silk handkerchief, diligently and carefully used, can at least make the shabbiness distinctly ashamed of itself. There are those, however, who seem to take a pride in shabbiness. One of the most successful, and deservedly successful, medical men in London tears round day by day, seated in a victoria, behind a pair of horses and a liveried coachman; and the whole turn-out, horses, man, victoria, and all, is so hopelessly and disreputably "seedy" as to suggest that a ten-pound note would be an excessive price for the lot. But the doctor himself, as he sits smiling and rubbing his hands in his carriage, looks so beaming, bland, and prosperous that the moment his face is seen, both carriage, horses, and hangdog coachman are alike forgotten. But it is not given to everyone to be so philosophically superior to his circumstances; and probably if Dr. Blank were as much below par in his purse, his practice, or his health as he is in his "coaching arrangements," he, too, would look as miserable and as hopeless as his turn-out.

Chemists who deal in powerful poisons and other dangerous substances use a weighing apparatus which is so finely balanced that the weight even of a single hair will "turn the scale." Few people understand that in modern life the scales of health and happiness may be as delicately balanced as the chemist's weighing apparatus. It is often said of a particular man or woman that he is a very good fellow, or that she is exceedingly pleasant, but "so very uncertain." Sometimes the spirits are overflowing even to boisterousness. At other times the melancholy is so deep that no prisoner condemned to be hanged could look more despairing. It is a question of "balance," not of balance of judgment or of moral worth, but of liver and stomach. Many a man groans *De Profundis* at bedtime who will sing *Te Deum Laudamus* in the morning solely as the result of two or three grains of hydrargyrum cum creta. That, of course, is a ridiculous aspect of the question, nevertheless it is perfectly true both in science and in experience. If a man be thoroughly below par his health and spirits are nothing but well-oiled weathercocks. The smallest puff of wind can turn south to north and summer into winter in a moment. "Now is the winter of our discontent made glorious summer by this sun of York" might be aptly parodied by, "Now is the midnight of our black despair made glorious sunrise by one small blue pill."

There is a justifiable object in presenting the thing in this absurd light. It is the order of the day to be below par in these times, and the writer is convinced that most persons who are below par ought to be thoroughly ashamed of themselves. There is a battle of life going on, no doubt; but the healthy soldier does not look profoundly miserable because he is in the midst of a campaign. In England, at the present time, there is a moderate degree of general prosperity. The bulk of the middle and upper classes, at any rate, are reasonably well off. They have, moreover, inherited fairly good constitutions from their progenitors. There is absolutely no reason why they should be below par, except that they have not brains and knowledge enough to keep themselves in health. It is a fact that the average well-doing workman is both healthier and happier than the average member of the middle and upper classes. He takes life more philosophically; he whistles and sings to a more advanced age; he has more courage, more hope; he is less easily broken down by reverses. Why is this? Because he has more stamina, more health, steadier health; he is less of a physiological weathercock; he neither despairs so quickly nor rejoices so precipitately as his heavier-pursed contemporary.

Stolidity used to be the distinguishing characteristic of John Bull, and a very fine characteristic it was. It threatens to be so no longer. Many of our fellow-mortals, and especially those of the gentler sex, do not know what it means. They live in a physiological condition which is persistently below par, with the result that their steadfastness of temper, of character, and even of outward bearing are gradually disappearing. No patriotic physician can contemplate such deterioration of national character without unhappiness. If it be said that science is cosmopolitan and knows nothing of patriotism, the answer is that British science at any rate knows how to be quite cosmopolitan and intensely British too. Biological science, as interpreted by the laureate, may be very careful of the type and very careless of the single life; but the same science as interpreted and made human by the physician's deeper instincts strives to preserve the single life, and thus to make sure that the type shall run no risk of extinction. Science, like Nature, is to be the servant of man as far as possible, as well as his master. If she shows us single lives everywhere deteriorating, and if she shows us at the same time the reason of the deterioration, it is not that we may fold our hands in feeble and useless despair, but that we may understand the true nature of the disease and set ourselves promptly to the discovery of a remedy.

The average Briton of the middle and upper classes, being so undoubtedly prosperous in his circumstances, can well afford to surround himself with a perfectly healthy environment, and can also arrange his business in such a way as to give himself the best possible chance of health and long life. Many persons will here demur. They will say that they themselves are little better than driven slaves. Their necessary expenses are represented by a certain round sum annually; and in order to make sure that that round sum shall be forthcoming at the right time, they must work quite as hard, and live in exactly the same way as they do now. That is to a large extent a pure delusion. Most families of the middle class spend at least a third more annually than they have any necessity to spend. If father and mother were skilful and economical in the use of money, and if they trained their sons and daughters to the same skill and economy, there could easily be saved a third of the annual outlay, in most families, that is now expended. That third would give all the margin that is required for the heads of the house and others to purchase health and pleasure with. That margin represents excessive work on the one hand, and on the other fresh air, change, amusement, and bodily exercise. The marginal "third" not being saved, but spent, the fresh air, the change, the amusement, and the bodily exercise are not secured, and so the physiological scale inevitably and persistently dips the wrong way. If we are asked to indicate how the money might be saved, we will put first and foremost on the list of extravagances the costly clothing worn by men and women, but particularly by women. Of course, we have not the slightest hope of altering the fashions of the times. All the same, it is quite certain that men and women could have all the warmth, comfort, and beauty in their clothing that they have now, and that for half the money, if they had but strength of mind to insist upon it. Similarly, large economies are practicable in house rents and in taxes, if we were resolute enough in insisting upon such economies. Social customs of other kinds waste enormous sums, the saving of which, if for no other reason than that it would put an end to the customs, would carry most of us a long way on the road to perfect health.

The plain truth is that in ultimate analysis the man who is below par mostly is so because there is a "screw loose" somewhere in his mental capacity. He is deficient in progressive energy, or in repressive steadfastness. He cannot say "yes" or "no" at the right time and in the right way. He is the slave of his business, or the slave of his wife, or of his social circumstances. As we often insist here, life is becoming more and more a "game of skill," a complicated chess problem; and it is only by carefully studying the rules of the game and resolutely putting them into practice, that any of us, even the best, can hope to win. What becomes of the worst is all too evident on every hand.

